# Macromolecular association creates dispersity of components that determines liquid–liquid phase separation of oppositely charged polyions

**DOI:** 10.1073/pnas.2628217123

**Published:** 2026-10-21

**Authors:** Matthias Ballauff, Matthew Tirrell

**Affiliations:** ^a^ Chemie und Biochemie, Freie Universität Berlin, Berlin 14195, Germany; ^b^ Pritzker School of Molecular Engineering, University of Chicago, Chicago, IL 60637

**Keywords:** liquid–liquid phase separation, polyelectrolyte complexation, biological condensates

## Abstract

Developing insight into liquid–liquid phase separation in complex polyelectrolyte mixtures has acquired new urgency considering the role this process plays in the formation of biomolecular condensates, which are involved in diverse processes including, stress response, signal transduction, metabolism, gene regulation, and many human diseases. Condensates can be viewed as a biological membraneless structure situated, in scale and function, between macromolecules and cells. The physical properties of, and the physico-chemical environments within, condensates are very important determinants of their biological function. These properties and environments are determined by the phase separation process. Earlier experimental work in one of our laboratories characterized in detail a phase separation process in solutions of oppositely charged polypeptides. Considering new information being developed on macromolecular association in homogeneous solutions of oppositely charged polyions, we have revisited our earlier data looking into the influence of intermolecular association on the outcome of the phase transition. Such influence is clearly established in this Brief Report. We demonstrate that dispersity of species in homogeneous solution owing to macromolecular association affects phase separation by creating an open miscibility gap, leading to a dependence of the phase compositions on the initial polymer volume fraction. It is very likely that this plays a significant role in the formation of condensates in the biological context that may have been overlooked.

Liquid–liquid phase separation (LLPS) on mixing highly charged polyelectrolytes of opposite charge in aqueous solution was first observed nearly a century ago (1). From the first effort at theoretical modeling seventy years ago (2), to recent efforts (3), the dominant approach has been to represent the polymer composition of such mixtures as a single variable, that is, treating the solution as a symmetric, stoichiometric mixture of two polyions of equal, monodisperse chain length, and equal charge fraction. This is a reasonable way to explore some aspects of this type of LLPS, not least because this precise situation can be approximated well, and LLPS behavior probed quantitatively, experimentally (4). Extensions of theory to investigate mixtures of asymmetric composition or charge fraction have also been undertaken (5, 6).

However, there is another type of composition effect that can strongly influence LLPS behavior. This derives from the increasingly recognized fact that very dilute solutions of oppositely charged polyions, or supernatant phases in LLPS, contain clusters of polyions. This fact has been noted by theorists (7, 8) and observed directly experimentally (9). This means that LLPS, even in systems of polyelectrolytes of low molecular dispersity, occurs in a mixture of multimolecular species with significant dispersity. The aim of this Brief Report is to demonstrate the effects of this clustering on experimental determination of LLPS phase boundaries *via* reexamination of previously published data from one of our laboratories (4).

## Results and Discussion

In 2018, Li et al. (4), published a detailed, quantitative study of the phase diagram of two highly charged polyelectrolytes of nearly equal and quite uniform chain length, *N*. Poly(L-lysine hydrochloride) (PLK; *N* = 20, 50, 100, and 400) and poly(D, L-glutamic acid) (PRE; *N* = 20, 50, 100, and 400) of the same length were mixed at a defined concentration of NaCl. Precise measurements of the volume fractions of the complexes in the dilute and the concentrated phase furnishes the full phase diagram as the function of the concentration of added salt. Thus, the salt concentration *c_s_* is the main variable, and phase separation takes only place below a critical value of *c_s_.* There are several features that are not compatible with a classical phase diagram for strictly binary mixtures: Li et al. determined the maximum salt concentration at which a phase transition can be seen. This “salt resistance” was found to decrease monotonically with the overall concentration of the polyelectrolytes. For a strictly binary system, however, the salt resistance should be given by the shape of the coexistence curve and should exhibit a maximum at the critical point. Moreover, the volume fraction of the dilute phase was found to depend markedly on the starting volume fraction of the polyelectrolytes.

**Fig. 1.**
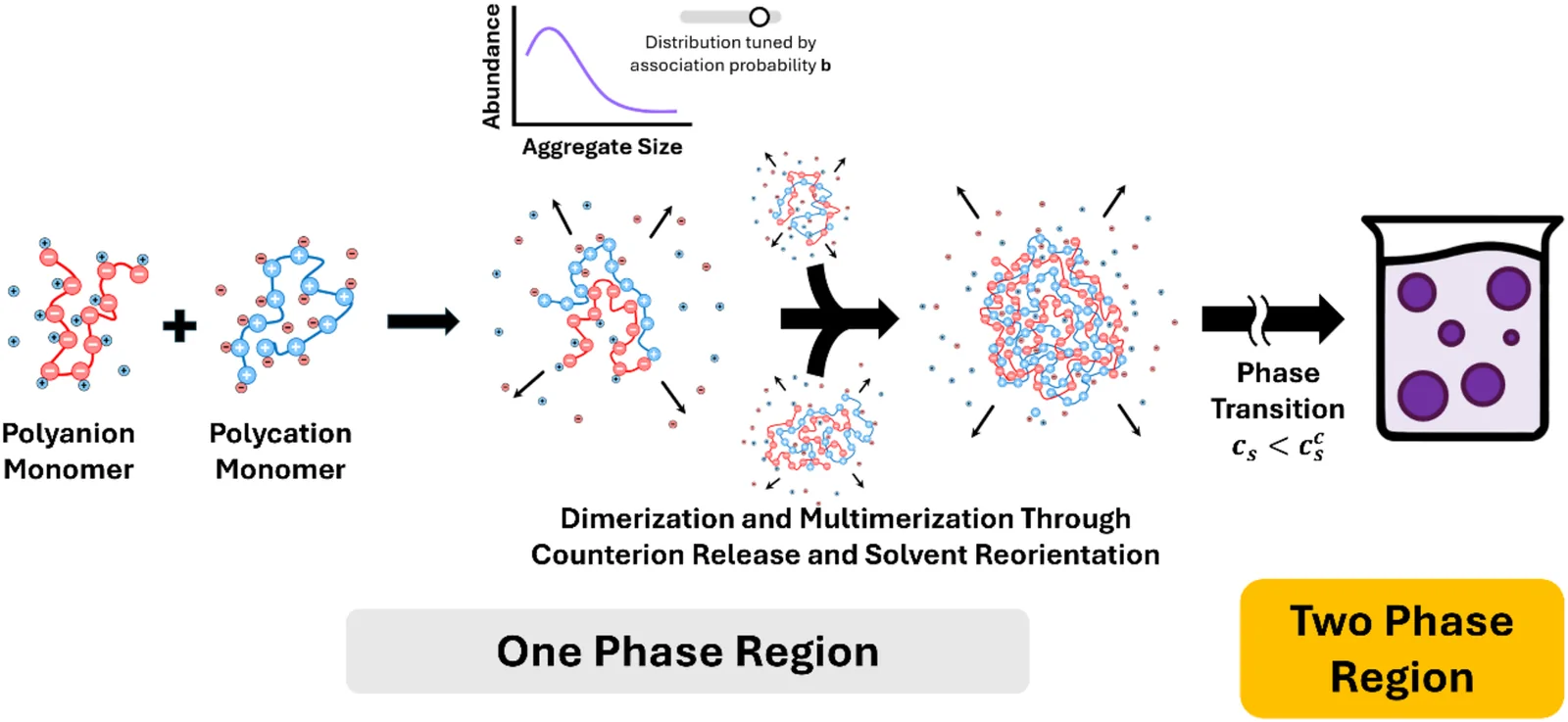
General mechanism of the liquid–liquid phase separation of highly charged polyelectrolytes. The anionic and the cationic polyelectrolytes form binary complexes which then associate to multimers of various sizes. The main driving force is the release of the counterions condensed to the polyelectrolytes. The formation of a solution of multimers takes place in the one-phase region and the complexes are stable even at low polymer concentrations or high salt concentrations. The solution of disperse multimers can phase separate if the salt concentration is lower than a critical salt concentration $c_{s}^{c}$.

Here, we demonstrate that the phase diagrams obtained by Li et al. (4) can be fully explained by the demixing of a disperse mixture of multimeric aggregates, as illustrated in Fig. 1: Association of positively and negatively charged polyelectrolytes leads to a distribution of clusters with a broad distribution in the homogeneous solution state. LLPS of this disperse mixture occurs below a critical salt concentration. Concomitantly, a fractionation takes place in which the larger aggregates will preferably be enriched in the concentrated phase.

**Fig. 2.**
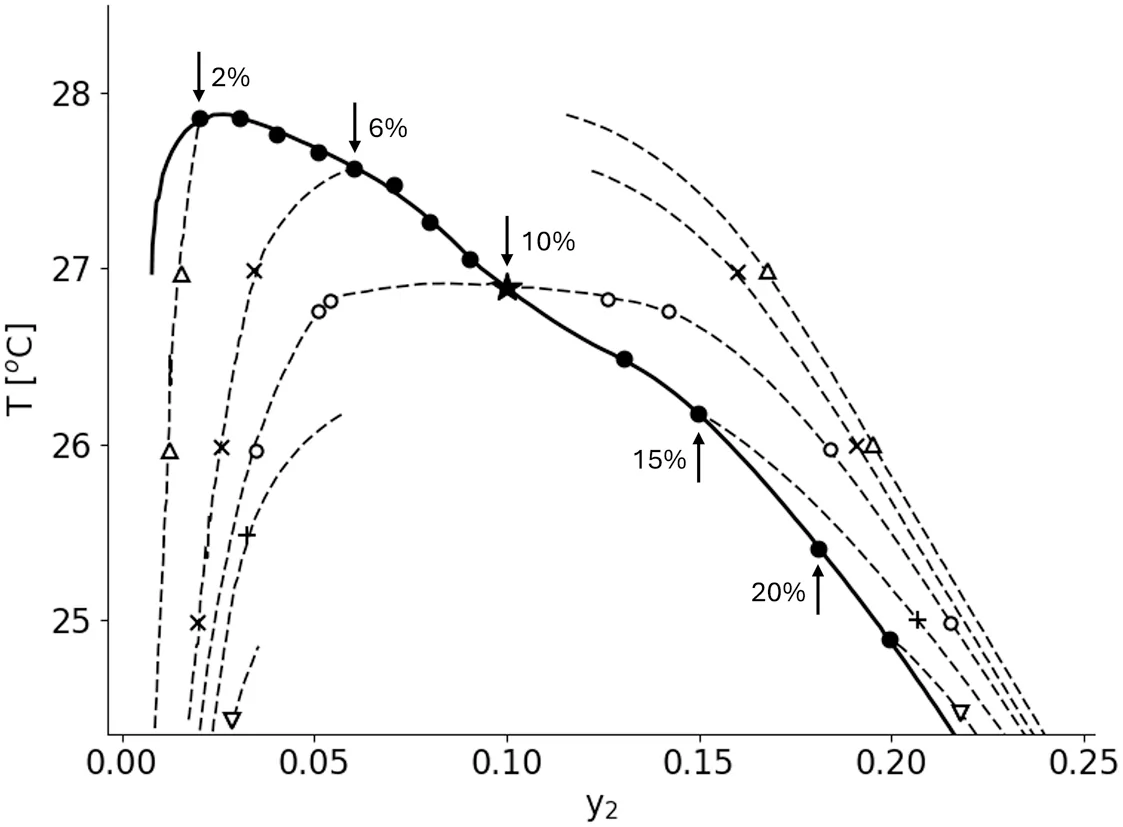
Phase equilibrium curves as a function of initial concentration (open symbols and ---) and the turbidity curve (● and **—**) for the polystyrene-cyclohexane system in the temperature range of 25 °C to 28 °C. M_n_ = 210,000. Cloud point line (●). Phase equilibria at the initial polymer concentrations, y_2_: △ 2%, X 6%, ◦ 10%, + 15%, ▽ 20%. Reproduced with permission from Rehage et al. (10). Image credit: Reproduced from ref. 10.

Phase separation in disperse mixtures of polymeric species was discussed in the most illuminating fashion by Rehage et al. (10) for the system polystyrene in cyclohexane. Figure 5 in that paper (10), reproduced below in Fig. 2, displays the main feature most clearly. In a disperse mixture, cloud point curves measured at different polymer concentrations do not map out a unique binodal, as they would in a strictly binary mixture. There is a line of polymer-volume-fraction-dependent transitions of the solution between turbid and clear, measured at different polymer volume fractions, which trace a cloud point curve, not a unique binodal. In the work of Rehage, et al, (10) cloud points were determined by varying temperature. As the temperature is further reduced below the cloud point, dilute and concentrated phases appear with an open miscibility gap between them. As the polymer volume fraction is increased from the dilute side, the supernatant concentrations increase and the concentrated phase concentrations decrease, as the miscibility gap closes at the critical point composition, then reopens.

**Fig. 3.**
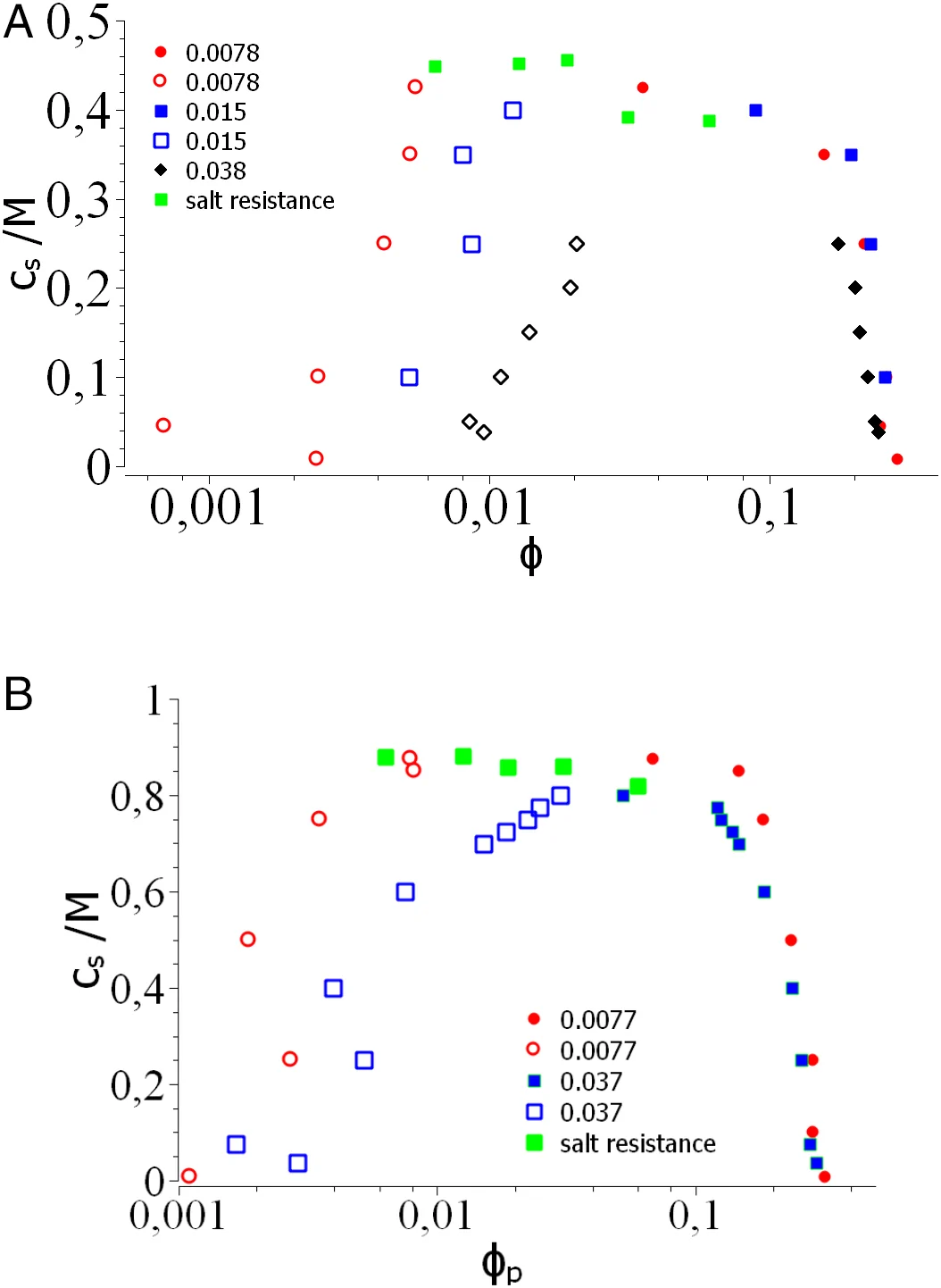
Phase diagram data replotted from reference 4. (*A*): N = 20, (*B*): N = 50. The total polymer volume fractions, φ, used in the experiment are given in the legends. Open symbols refer to the dilute branch, filled symbols refer to the concentrated phase. The 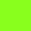 symbols are the experimental salt resistance which defines the cloud point lines.

The physics leading to these initial-concentration-dependent curves results directly from the dispersity of the polymer mixture in solution. As the control variable, which is temperature in Fig. 2, is reduced from full miscibility toward phase separation, the species with higher molecular weight will be enriched in the concentrated phase whereas the dilute phase will preferably contain the lower molecular weights. This leads to the open miscibility gap. Only at the critical composition, 10% in Fig. 3, is there a closed binodal curve.

In LLPS of charged polyelectrolytes, salt concentration plays the role of control variable that temperature plays in Fig. 2. All other features of the LLPS agree directly with the findings shown in Fig. 2. Dispersity in size of the clusters of associated polyions produces a similar open miscibility gap in the phase diagrams. This is seen in Fig. 3, where selected data for N = 50 and for N = 20 taken from the work of Li et al. (4) are plotted. Moreover, the composition of the dilute phase depends markedly on the overall volume fraction whereby the miscibility gap becomes smaller with increasing total polymer concentration (self-suppression, cf. ref. 4). Comparison of Fig. 2 with Fig. 3 now demonstrates that the salt resistance (green squares in Fig. 3) corresponds to the cloud point curve (solid line in Fig. 2). It marks the salt concentration where the first droplet of the concentrated phase is formed. For strictly binary mixture this line would coincide with bell-shaped top of the diagram. All these experimental features cannot be reconciled with the classical phase diagram of a binary mixture but are in full agreement with expectations for the de-mixing of a disperse mixture as shown in Fig. 2.

Formation of biomolecular condensates in cells takes place under more chemically diverse and heterogeneous conditions than the ones we have analyzed here which suggests that the effects we are pointing out here may be even more important in biology than in our simplified physical chemistry system. It is also pertinent to point out that heterogeneous distributions of clusters have been observed in dilute solutions of certain proteins undergoing simple coacervation (rather than complex coacervation) (11, 12), so the effects of species dispersity in dilute solution on LLPS may be more general than the polyelectrolyte complexation-driven LLPS that we have studied. Though the experimental facts speak for themselves, we are developing a modeling approach based on the Flory–Huggins theory, augmented by some assumptions about interaction parameters and the nature of the cluster distribution, that may provide a tool to accelerate learning about dispersity effects in solutions of oppositely charged polyions. What is unambiguous is the appearance of an open miscibility gap and initial-concentration-dependent phase boundaries in LLPS in these solutions of oppositely charged polypeptides.

## Supplementary Material

Dataset S01 (PDF)

## Data Availability

All study data are included in the article and/or *SI Appendix*. Previously published data were used for this work (4).
