# Supplementary material for: Macromolecular association creates dispersity of components that determines liquid–liquid phase separation of oppositely charged polyions
Source: Proc Natl Acad Sci U S A. 2026 Oct 21;123(43):e2628217123. doi: 10.1073/pnas.2628217123 (PMC13715835; doi:10.1073/pnas.2628217123)
Supplement: Supplementary file 1 [file pnas.2628217123.sd01.pdf]

**Polymer density**	1.2515	This is for the PRE+PLKC system
**Counterion density**	2.16	
**water density**	1	

N=100

DATA

Initial Polymer concentration (wt%)	Added Salt conc. (M)	Coacervate polymer conc.(wt. fr.)	Coacervate counterion conc. (wt. fr.)	Supernatant polymer conc. (wt. fr.)	Supernatant counterion conc. (wt. fr.)
0.00990099	0	0.37419875	0.0027939	0.00065651	0.00295122
0.009843973	0.1	0.36348535	0.00649351	0.00067224	0.0072277
0.009759668	0.25	0.33224326	0.01178482	0.00066463	0.01424677
0.009622324	0.5	0.28461716	0.0188619	0.00234443	0.02645544
0.009488791	0.75	0.22863995	0.0305134	0.00607871	0.03847597
0.009358914	1	0.02402158	0.05035518	0.00851874	0.05144579
0.019607843	0	0.367693	0.00474	0	0.00447824
0.019330675	0.25	0.30739268	0.00984111	0.00045014	0.01576596
0.018799201	0.75	0.20654294	0.03247016	0.00623639	0.04052598
0.018594705	0.95	0.06082919	0.04568277	0.01425125	0.04989371
0.029126214	0	0.35760737	0.00418474	0.0017096	0.00427177
0.028321926	0.5	0.25549738	0.02236779	0.00643732	0.02774634
0.027709786	0.9	0.08742367	0.04464314	0.02231678	0.04851828
0.047619048	0	0.34548694	0.00668353	0.00421363	0.0073188
0.047355211	0.1	0.32923701	0.0128093	0.00413874	0.0155226
0.046964894	0.25	0.30601239	0.01463159	0.0040519	0.02108694
0.046328469	0.5	0.255575536	0.02766262	0.00722191	0.03394116
0.045709062	0.75	0.18773467	0.040463115	0.01512674	0.04608579
0.045465912	0.85	0.11571735	0.04324651	0.02725658	0.04755917
0.090909091	0	0.32863089	0.01295881	0.0103445	0.01391
0.08855435	0.5	0.21137923	0.0352692	0.01808106	0.03944781
salt resistance		0.007623582	0.056484982		
		0.015146869	0.055117873		
		0.022541379	0.055045126		
		0.037035608	0.053646153		
		0.071578365	0.049706317		

STD DEV

Coacervate polymer conc.(wt. fr.)	Coacervate counterion conc. (wt. fr.)	Supernatant polymer conc. (wt. fr.)	Supernatant counterion conc. (wt. fr.)
0.005172577	0.002326896	0.000288896	0.000861235
0.002776318	0.000213563	0.00029131	0.000353268
0.002261397	0.000294788	0.001692019	0.000645033
0.005656908	0.001013299	0.000580905	0.000453078
0.000719978	0.000411194	0.001094408	0.000919559
0.002577125	0.000392778	6.01727E-05	0.000323908
0.005129039	0.00160825	0	0.000195436
0.00657696	0.000147541	0.000390018	0.000648701
0.002101322	0.001268402	0.000852365	0.000627231
0.018448637	0.003726518	0.000364728	0.000624415
0.001936378	0.000851398	0.000791392	0.000318222
0.002763117	5.32903E-05	0.000383834	0.00027553
0.016750758	0.001098304	0.001185979	0.000814992
0.007783587	0.002832583	0.000745777	0.002287417
0.013063109	0.00025133	0.000495412	0.000325227
0.002424225	0.000794588	0.000537407	0.000612502
0.004370048	0.000816232	0.000585587	0.000489427
0.005847858	0.000749495	0.00062749	0.001078135
0.016796941	0.00274083	0.000452401	0.000750101
0.005075681	0.000826282	0.000546316	0.000835145
0.003426828	0.000743368	0.000781849	0.000498153

DATA				STD DEV			
Coacervate polymer conc.(vol. fr.)	Coacervate counterion conc. (vol. fr.)	Supernatant polymer conc. (vol. fr.)	Supernatant counterion conc. (vol. fr.)	Coacervate polymer conc.(vol. fr.)	Coacervate counterion conc. (vol. fr.)	Supernatant polymer conc. (vol. fr.)	Supernatant counterion conc. (vol. fr.)
0.323838264	0.001400921	0.000525481	0.001368655	0.004142585	0.001079738	0.00023096	0.000398928
0.314510108	0.0032554	0.000539314	0.003359652	0.002219885	9.89383E-05	0.000232826	0.000163591
0.286411611	0.005886193	0.000535233	0.006647475	0.001808057	0.00013656	0.001352921	0.000298832
0.243837389	0.009362714	0.001901203	0.01243035	0.004527713	0.00046991	0.000464334	0.000209834
0.194837755	0.015065679	0.004965814	0.0182115	0.000575503	0.000190437	0.000875102	0.000426026
0.019826087	0.024080012	0.007012574	0.024537429	0.002060731	0.000181974	4.80894E-05	0.000149985
0.318117712	0.002376063	0	0.002078257	0.004106092	0.000745974	0	9.04891E-05
0.263274121	0.004883552	0.000362785	0.007362055	0.005262634	6.8402E-05	0.000311774	0.000300453
0.175373583	0.015974062	0.005100532	0.019204051	0.001680897	0.000587872	0.000681421	0.000290532
0.050459802	0.021956497	0.011735391	0.023804964	0.014825857	0.001735145	0.000291552	0.000289199
0.308614994	0.002092455	0.001369653	0.001982902	0.001548556	0.0003945	0.000632564	0.000147374
0.217962313	0.011055926	0.005228354	0.013056978	0.002209134	2.46859E-05	0.000306768	0.000127589
0.072882928	0.021563965	0.018393788	0.023169792	0.013437705	0.000510493	0.000948287	0.000377566
0.29780327	0.003337958	0.003383025	0.003404598	0.006238655	0.00131544	0.000596729	0.001060451
0.283803543	0.00639752	0.003337623	0.007252883	0.010466851	0.000116678	0.000395964	0.000150609
0.262738343	0.007278687	0.003277419	0.009882432	0.001938828	0.000368201	0.000429598	0.00028369
0.218696587	0.013714916	0.005886442	0.016028933	0.003496451	0.000378383	0.000468086	0.000226673
0.15949058	0.01991714	0.012432379	0.021945882	0.004680063	0.000347537	0.000501744	0.000499489
0.096970034	0.020997483	0.02247631	0.022722966	0.013486823	0.001275084	0.001961326	0.00034758
0.283268379	0.006471902	0.008345371	0.006501901	0.004061623	0.000383099	0.000436772	0.000386857
0.179953327	0.017396836	0.014815202	0.018727667	0.002741159	0.000344527	0.000624995	0.000230724
0.006292062	0.027011209						
0.012511397	0.026378644						
0.018647194	0.026383292						
0.030706153	0.025770427						
0.059644148	0.023997983						

N=20

DATA

Initial Polymer concentration (wt%)	Added Salt conc. (M)	Coacervate polymer conc.(wt. fr.)	Coacervate counterion conc. (wt. fr.)	Supernatant polymer conc. (wt. fr.)	Supernatant counterion conc. (wt. fr.)
0.00990099	0	0.33253038	0.00101833	0.00303789	0.00139455
0.009875251	0.045	0.29338604	0.00395452	0.00086499	0.00483317
0.009843973	0.1	0.30779743	0.00472547	0.00306648	0.00664403
0.009759668	0.25	0.25739264	0.01091512	0.00530188	0.01435805
0.009704263	0.35	0.18782999	0.0190604	0.00653927	0.02124191
0.009663119	0.425	0.04388292	0.02576	0.00681456	0.0266
0.019496028	0.1	0.30326792	0.00584534	0.00645539	0.00644987
0.01934526	0.25	0.27017078	0.0121479	0.01079634	0.01315941
0.01922199	0.35	0.233263093	0.018394544	0.009983191	0.021674095
0.019168104	0.4	0.108663829	0.023623765	0.015065149	0.02445853
0.02871844	0.25	0.25386585	0.0107105	0.01564949	0.01125867
0.028558509	0.35	0.1656589	0.0223702	0.02122507	0.022846787
0.047619048	0	0.28831816	0.00941741	0.01194948	0.01024462
0.047552813	0.05	0.28064132	0.0110067	0.01057158	0.01296122
0.047355211	0.1	0.26474022	0.01148368	0.0136868	0.01417944
0.047224387	0.15	0.24956895	0.01279694	0.01730473	0.01614597
0.047094283	0.2	0.24114782	0.01470916	0.02426893	0.0172875
0.046964894	0.25	0.21081942	0.0212591	0.02545174	0.02156546
0.090909091	0	0.22465183	0.01307792	0.03511651	0.01729894
salt resistance		0.007871381	0.025816669		
		0.015613948	0.025980838		
		0.023230839	0.026142341		
		0.038256649	0.022445477		
		0.073660957	0.022057269		

STD DEV

Coacervate polymer conc.(wt. fr.)	Coacervate counterion conc. (wt. fr.)	Supernatant polymer conc. (wt. fr.)	Supernatant counterion conc. (wt. fr.)
0.001194047	0.001763799	0.000505689	0.000505945
0.000999334	5.5284E-05	0.000588738	0.000265048
0.001049494	0.00032693	1.30257E-05	2.82223E-05
0.028437596	0.000937206	0.000339	0.000155441
0.029913872	0.001905266	0.00039224	0.000357307
0.012216865	0.001544689	0.000278111	0.000311669
0.001476713	0.002223328	0.001578436	0.0011631
0.005175007	0.000487039	0.000586204	0.001042019
0.005782137	0.00230016	0.001186901	0.00050102
0.024277648	0.001012543	0.001338918	0.000421808
0.004017513	0.002928038	0.000459068	0.001830478
0.022100572	0.001687209	0.001727116	0.00143622
0.002571144	0.000903705	0.00151841	0.001002296
0.000280786	0.00080538	0.000358477	0.000497552
0.00107947	0.000669735	0.000496565	0.000500471
0.000912564	9.31409E-05	0.000247707	0.000543622
0.000601042	1.59689E-05	0.000315517	0.000305622
0.002376699	0.000239667	0.001307179	0.001115342
0.001168441	9.87039E-05	0.00087768	0.000515709

DATA				STD DEV			
Coacervate polymer conc.(vol. fr.)	Coacervate counterion conc. (vol. fr.)	Supernatant polymer conc. (vol. fr.)	Supernatant counterion conc. (vol. fr.)	Coacervate polymer conc.(vol. fr.)	Coacervate counterion conc. (vol. fr.)	Supernatant polymer conc. (vol. fr.)	Supernatant counterion conc. (vol. fr.)
0.284899661	0.000505506	0.002430703	0.000646504	0.000955226	0.000817544	0.000404218	0.000234321
0.249678438	0.001949901	0.000693082	0.002243793	0.000798693	2.56004E-05	0.000470548	0.00012274
0.26286961	0.002338285	0.002460539	0.003088865	0.000838913	0.000151415	1.04082E-05	1.30661E-05
0.218234834	0.005362083	0.004273929	0.0067061	0.022864986	0.000436607	0.000270916	7.19743E-05
0.157648205	0.009269007	0.005292476	0.009960939	0.024071752	0.000888317	0.000313501	0.000165465
0.03587697	0.012202342	0.005531711	0.012510665	0.009793948	0.00071749	0.000222272	0.000144323
0.258915787	0.002891472	0.005182798	0.003000336	0.001181716	0.001030855	0.001262424	0.00053898
0.22985679	0.005988213	0.008707145	0.006149117	0.004140433	0.000225775	0.000468718	0.000482743
0.197601659	0.009028398	0.00808734	0.010173126	0.004631268	0.001067448	0.000948864	0.000232071
0.089931653	0.011328014	0.01223543	0.011509416	0.019504605	0.000471326	0.001070381	0.000195378
0.215057773	0.005256997	0.012620585	0.0052607	0.003217816	0.001358807	0.000367209	0.000848356
0.138649698	0.010848036	0.017244847	0.01075505	0.017754205	0.000785315	0.001381582	0.000665661
0.245867108	0.004653042	0.009624187	0.004780662	0.002056511	0.000418802	0.001214296	0.000464417
0.239144676	0.005434297	0.008524574	0.00605558	0.00022447	0.000373044	0.000286535	0.000230426
0.224889826	0.005652077	0.011050863	0.006633312	0.000863039	0.000310241	0.000396922	0.000231785
0.211475353	0.006282789	0.013997237	0.007566913	0.000729347	4.31309E-05	0.000197995	0.000251763
0.204195522	0.00721652	0.019670428	0.008118438	0.00048032	7.39397E-06	0.000252168	0.000141524
0.178028278	0.010401605	0.020682304	0.010153535	0.001900232	0.000111024	0.00104539	0.000516807
0.189386163	0.00638784	0.028525853	0.008141865	0.000933901	4.57094E-05	0.000701621	0.000238863
0.006388232	0.012139675						
0.012693118	0.012237307						
0.018916279	0.012333672						
0.031184279	0.010600704						
0.060469552	0.010491274						

N=400

DATA

Initial Polymer concentration (wt%)	Added Salt conc. (M)	Coacervate polymer conc.(wt. fr.)	Coacervate counterion conc. (wt. fr.)	Supernatant polymer conc. (wt. fr.)	Supernatant counterion conc. (wt. fr.)
0.00990099	0	0.37840432	0.00050993	0.00050463	0.00050463
0.009832648	0.12	0.35795805	0.00861409	0.00050387	0.0093566
0.009759668	0.25	0.3418939	0.01137973	0.00182837	0.01478865
0.009622324	0.5	0.30073423	0.02052521	0.00249836	0.02792928
0.009488791	0.75	0.248249	0.02940154	0.00317524	0.03861288
0.009358914	1	0.16615309	0.04410401	0.00478026	0.05062477
0.009307954	1.1	0.12795085	0.05242621	0.00704084	0.05780196
0.009312201	1.16	0.1235125	0.05746024	0.00908004	0.05948441
0.009245434	1.19	0.06324401	0.06138875	0.00908648	0.06144678
0.019607843	0	0.36861512	0.0031818	0.00068212	0.00320905
0.019330675	0.25	0.3312132	0.0129293	0.00301065	0.01538027
0.019061234	0.5	0.29856504	0.02039511	0.00251226	0.02864047
0.018544274	1	0.14740307	0.04873828	0.00774109	0.0545122
0.029126214	0	0.35468077	0.00590451	0.00134418	0.0057152
0.02871844	0.25	0.33355801	0.01433704	0.00166576	0.01817085
0.028321926	0.5	0.30291602	0.02330299	0.00286316	0.03047045
0.02808923	0.65	0.25616464	0.0268253	0.00410883	0.03746155
0.027936212	0.75	0.23896979	0.03385049	0.00587856	0.04294687
0.027560864	1	0.15108207	0.04834257	0.00882955	0.05525568
0.047619048	0	0.35198797	0.00846822	0.00268076	0.00871466
0.047355211	0.1	0.34559603	0.01040888	0.0025214	0.01294797
0.046964894	0.25	0.32514746	0.0151268	0.00283492	0.01983886
0.046328469	0.5	0.27831898	0.02556001	0.00476947	0.03421499
0.045709062	0.75	0.21095448	0.03861148	0.00684056	0.04674156
0.045165588	0.975	0.12269242	0.05166348	0.0167663	0.05762554
salt resistance		0.007551232	0.065439185		
		0.015024295	0.062764242		
		0.022420396	0.060116847		
		0.036790971	0.059897262		
		0.071121367	0.055773544		

STD DEV

Coacervate polymer conc.(wt. fr.)	Coacervate counterion conc. (wt. fr.)	Supernatant polymer conc. (wt. fr.)	Supernatant counterion conc. (wt. fr.)
0.003680697	0.000451551	1.91178E-06	1.91178E-06
0.003418274	7.43028E-05	1.25407E-06	3.22213E-06
0.003171253	0.001775138	0.000292208	0.001026861
0.002720684	0.00111537	0.000539723	0.000807931
0.004189858	0.000454401	0.000281545	0.001458373
0.010309179	0.002111348	0.000735525	0.000843267
0.004768782	0.001775195	0.000746919	0.000679383
0.001578643	0.001203855	0.000559025	7.20548E-05
0.008811144	0.001074197	0.000332663	0.000247708
0.004257039	0.000474269	0.000313263	0.000721634
0.006314708	0.001190747	0.000508921	0.00048688
0.000505105	0.001228203	2.0933E-05	0.001369615
0.009193589	0.001266898	0.000308433	0.000691302
0.016573351	0.000353214	0.000287556	0.00028912
0.00426692	0.00076565	0.000758843	0.000626456
0.002263888	0.000256708	0.000776569	0.000704332
0.003175655	0.002095703	0.000761443	0.001236211
0.001283533	0.000682477	2.17623E-05	0.00044032
0.002369831	0.00066835	0.002146467	0.000216778
0.002765268	0.001676658	0.000287006	0.00079414
0.002235449	0.001734892	0.000499054	0.000299466
0.001625085	0.00118478	0.000767311	0.001420024
0.002724982	0.001458192	0.000555344	0.000661705
0.004640951	0.000822902	4.16524E-05	0.000281795
0.005519919	0.000937234	0.001211078	0.00121089

DATA				STD DEV			
Coacervate polymer conc.(vol. fr.)	Coacervate counterion conc. (vol. fr.)	Supernatant polymer conc. (vol. fr.)	Supernatant counterion conc. (vol. fr.)	Coacervate polymer conc.(vol. fr.)	Coacervate counterion conc. (vol. fr.)	Supernatant polymer conc. (vol. fr.)	Supernatant counterion conc. (vol. fr.)
0.327342609	0.000255584	0.00040337	0.000233712	0.00294392	0.000209257	1.52759E-06	8.85085E-07
0.30973696	0.004318644	0.000404687	0.004354079	0.002733328	3.44244E-05	1.00206E-06	1.49173E-06
0.295279499	0.00569444	0.001473184	0.006903966	0.002537999	0.000823133	0.000233629	0.000475689
0.258791768	0.010233691	0.002027724	0.013133811	0.002176432	0.000516967	0.000431495	0.000374245
0.212304827	0.014568658	0.002592563	0.018266779	0.003351509	0.0002106	0.000225155	0.00067574
0.140799338	0.021654461	0.003930253	0.024116217	0.008263949	0.000980619	0.000588068	0.000390636
0.108058874	0.025653276	0.005814645	0.02765785	0.003817752	0.000823423	0.000597126	0.000314691
0.10451064	0.028170473	0.007508902	0.028501578	0.001262618	0.000557878	0.000446751	3.33637E-05
0.052953341	0.02978104	0.007522442	0.029474022	0.007057034	0.000498484	0.000265864	0.000114703
0.318690692	0.001593845	0.000546058	0.00148844	0.00340533	0.000219813	0.000250423	0.000334241
0.285649265	0.00646067	0.002427149	0.007184182	0.00505536	0.000552326	0.000406796	0.00022549
0.256785235	0.010163288	0.002039803	0.013473515	0.000403907	0.000569046	1.67387E-05	0.00063455
0.124741206	0.023897403	0.006382218	0.02603996	0.007364673	0.000588013	0.000246557	0.000320186
0.306200278	0.002953445	0.001077654	0.002654791	0.013289572	0.000164103	0.000229818	0.00013388
0.288053056	0.007173609	0.001344582	0.008498204	0.003413776	0.000354918	0.000606643	0.000290168
0.26121227	0.011642871	0.002327203	0.014349762	0.001810013	0.000118917	0.000620842	0.000326254
0.219122945	0.013295062	0.003353357	0.017714318	0.002541962	0.000971947	0.000608921	0.000572788
0.204483955	0.016782563	0.004813927	0.020376855	0.001026236	0.000316159	1.73932E-05	0.000203901
0.127925969	0.023716611	0.007284254	0.026411956	0.001895175	0.00030968	0.001716055	0.000100415
0.304150162	0.004239645	0.002153275	0.004055731	0.002212785	0.000777362	0.000229441	0.000367836
0.298548704	0.005209878	0.002029845	0.006039487	0.001788685	0.000804301	0.000398869	0.000138678
0.280406219	0.007558427	0.002290931	0.009288915	0.001299761	0.000549038	0.000613676	0.000658022
0.239039148	0.012719333	0.003886134	0.016152553	0.002180274	0.000675988	0.00044395	0.000306488
0.179919438	0.019080198	0.005614543	0.022228137	0.003713415	0.000381497	3.32873E-05	0.000130481
0.103457609	0.025240936	0.013873036	0.027626534	0.004417766	0.000434606	0.000968567	0.000561099
0.006263365	0.031448858						
0.012462736	0.030165373						
0.01859909	0.028894934						
0.030608383	0.028872422						
0.059459688	0.027016425						

N=50

DATA

Initial Polymer concentration (wt%)	Added Salt conc. (M)	Coacervate polymer conc.(wt. fr.)	Coacervate counterion conc. (wt. fr.)	Supernatant polymer conc. (wt. fr.)	Supernatant counterion conc. (wt. fr.)
0.00990099	0	0.36797472	0.00133583	0.00137894	0.00137894
0.009843973	0.1	0.33160876	0.00649597	0.00051485	0.007094676
0.009759668	0.25	0.33077307	0.010723861	0.00339599	0.01341442
0.009622324	0.5	0.2774263	0.01991609	0.00233149	0.02718897
0.009488791	0.75	0.21784166	0.03703727	0.00439117	0.04125585
0.00943641	0.85	0.17686618	0.0408246	0.01022472	0.04417078
0.009423405	0.875	0.08394225	0.04334843	0.00986877	0.04703918
0.019607843	0	0.36546302	0.00419218	0.0031481	0.00202345
0.019330675	0.25	0.32766005	0.01388938	0.00292789	0.01352831
0.029126214	0	0.35272959	0.00543197	0.00393144	0.00404965
0.02871844	0.25	0.31399406	0.01679753	0.00564183	0.01804566
0.047619048	0	0.34214061	0.00896869	0.00360458	0.00924611
0.047420896	0.075	0.32373222	0.01212373	0.0020752	0.01313634
0.046964894	0.25	0.302833	0.01654244	0.00650894	0.02090845
0.046580958	0.4	0.27919805	0.02452038	0.00494974	0.03036373
0.046078702	0.6	0.22077707	0.03196928	0.00937844	0.03972695
0.045831615	0.7	0.17700731	0.03884508	0.0188974	0.04429901
0.045770256	0.725	0.16783502	0.04184906	0.02298565	0.04511618
0.045709062	0.75	0.15163793	0.04360231	0.02771598	0.0464749
0.045660224	0.775	0.14770452	0.0381388	0.0309902	0.04701827
0.045587163	0.8	0.06457871	0.04406878	0.03718952	0.04713485
salt resistance		0.007676115	0.049983347		
		0.015229703	0.049950601		
		0.022694456	0.04862798		
		0.037233673	0.04858509		
		0.071855394	0.046028409		

STD DEV

Coacervate polymer conc.(wt. fr.)	Coacervate counterion conc. (wt. fr.)	Supernatant polymer conc. (wt. fr.)	Supernatant counterion conc. (wt. fr.)
0.001710591	0.000559747	1.68055E-05	1.68055E-05
0.000746255	0.000178962	1.18042E-05	0.000506911
0.010385313	9.15195E-05	1.03973E-05	0.000364478
0.005432323	0.000485386	0.000363696	0.000699378
0.00206948	0.000896463	0.001022193	0.000760807
0.01398768	0.004745643	0.000275449	0.000939643
0.005559391	0.001609695	8.26374E-05	7.1313E-05
0.00339043	0.000493341	0.000395831	1.42479E-05
0.002147838	0.000495049	0.000352544	0.00023111
0.003672703	0.000211769	0.000399145	0.000614748
0.006603409	0.00071835	0.001054572	0.000321896
0.002669903	7.96307E-05	0.000352469	0.000486085
0.005355103	0.000136095	0.000526449	0.000166912
0.00268834	0.000929868	0.000938877	0.000392666
0.005399919	0.000654973	0.00041175	0.000482465
0.000877689	0.000492003	0.000901924	0.000797987
0.006603409	0.00071835	0.001054572	0.000321896
0.003767794	0.000686646	0.001952631	0.000756431
0.006603409	0.00071835	0.001054572	0.000321896
0.001783325	0.0006815	0.000667672	0.00066737
0.005095559	0.00195983	0.00075523	0.000233244

DATA				STD DEV			
Coacervate polymer conc.(vol. fr.)	Coacervate counterion conc. (vol. fr.)	Supernatant polymer conc. (vol. fr.)	Supernatant counterion conc. (vol. fr.)	Coacervate polymer conc.(vol. fr.)	Coacervate counterion conc. (vol. fr.)	Supernatant polymer conc. (vol. fr.)	Supernatant counterion conc. (vol. fr.)
0.317751946	0.000668342	0.001102952	0.000639048	0.001367714	0.000259309	1.34285E-05	7.78043E-06
0.28495226	0.003234203	0.000413003	0.003297477	0.000596435	8.28729E-05	9.43465E-06	0.000234746
0.284878307	0.005351278	0.002735106	0.006259747	0.008316057	4.24608E-05	8.3095E-06	0.000168773
0.237453061	0.009876688	0.001891461	0.012780082	0.004346528	0.00022502	0.000290738	0.000323931
0.185900274	0.018312817	0.003591467	0.019550338	0.001655085	0.000415402	0.000817276	0.000352442
0.149939979	0.020052646	0.008386134	0.020990488	0.011236957	0.002208896	0.000220218	0.000435264
0.069878854	0.020908152	0.008106413	0.022387341	0.004451003	0.000746709	6.60343E-05	3.30171E-05
0.315934456	0.002099764	0.002519794	0.000938396	0.002711659	0.000228615	0.000316313	6.59682E-06
0.282524399	0.006938929	0.002358024	0.006312684	0.001717409	0.000229349	0.000281752	0.000107016
0.304303448	0.002715185	0.003150724	0.001880413	0.002937143	9.81247E-05	0.000319064	0.000284722
0.270395295	0.008381085	0.004557388	0.008445899	0.005285448	0.00033314	0.00084297	0.000149083
0.295095392	0.004481918	0.00289669	0.004305102	0.002134599	3.68874E-05	0.000281731	0.000225114
0.27861523	0.006045501	0.001670653	0.006127421	0.004283871	6.30794E-05	0.000420737	7.72892E-05
0.260116802	0.008232692	0.005266941	0.009802731	0.00215033	0.000430943	0.000750501	0.000181862
0.23969582	0.012196982	0.004024677	0.014304771	0.004320966	0.000303665	0.000329118	0.00022344
0.187977296	0.015771078	0.007671897	0.018829314	0.000701619	0.00022788	0.000721114	0.000369664
0.149895068	0.019059402	0.015528189	0.021090646	0.005285448	0.00033314	0.000842971	0.000149083
0.142093063	0.020528305	0.01891206	0.021507576	0.003014016	0.00031825	0.00156148	0.000350479
0.128066317	0.021336033	0.022843589	0.022193698	0.005285448	0.00033314	0.000842971	0.000149083
0.124255186	0.018589378	0.025567259	0.022475199	0.001425983	0.000315738	0.00053376	0.00030912
0.053563853	0.021178275	0.030723272	0.022561404	0.004080033	0.000909217	0.000603627	0.000108013
0.006312721	0.023816481						
0.012544049	0.023837688						
0.018707675	0.023225409						
0.03078482	0.023274526						
0.059755373	0.022177912						
